# A one-step cloning method for the construction of somatic cell gene targeting vectors: application to production of human knockout cell lines

**DOI:** 10.1186/1472-6750-12-71

**Published:** 2012-10-09

**Authors:** Yi Liu, Shangze Li, Huihui Zhang, Zurong Wan, Xiaodong Zhang, Runlei Du

**Affiliations:** 1 College of Life Sciences, Wuhan University, Wuhan, Hubei, PR China

## Abstract

**Background:**

Gene targeting is a powerful method that can be used for examining the functions of genes. Traditionally, the construction of knockout (KO) vectors requires an amplification step to obtain two homologous, large fragments of genomic DNA. Restriction enzymes that cut at unique recognitions sites and numerous cloning steps are then carried out; this is often a time-consuming and frustrating process.

**Results:**

We have developed a one-step cloning method for the insertion of two arms into a KO vector using exonuclease III. We modified an adeno-associated virus KO shuttle vector (pTK-LoxP-NEO-AAV) to yield pAAV-LIC, which contained two cassettes at the two multiple-cloning sites. The vector was digested with *Eco*RV to give two fragments. The two homologous arms, which had an overlap of 16 bases with the ends of the vector fragments, were amplified by polymerase chain reaction. After purification, the four fragments were mixed and treated with exonuclease III, then transformed into *Escherichia coli* to obtain the desired clones. Using this method, we constructed SirT1 and HDAC2 KO vectors, which were used to establish SirT1 KO cells from the colorectal cancer cell line (HCT116) and HDAC2 KO cells from the colorectal cancer cell line (DLD1).

**Conclusions:**

Our method is a fast, simple, and efficient technique for cloning, and has great potential for high-throughput construction of KO vectors.

## Background

Gene targeting is a powerful method for the production of genetically modified cell lines or animals, allowing for the various functions of genes to be studied. In mice, several thousand genes have been disrupted using homologous recombination. However, when these methods have been applied to human somatic cells they have generally been ineffective because of very low targeting efficiencies [1]. Although RNA interference can reduce the expression of a gene, interpretation of such experiments can be unreliable because of non-specific targeting or incomplete inactivation of genes. Therefore, we believe it is essential to improve the efficiency of knockout (KO) approaches in human somatic cells.

Recombinant adeno-associated viruses (rAAVs), such as human parvovirus, possess a single-stranded DNA genome of around 4.7 kb. Hirata [2] and Porteus [3] found that rAAVs can be used to target genes in human cell lines. Use of these rAAVs resulted in higher targeting frequencies than those obtained with conventional plasmid vectors [4,5]. The wild-type human parvovirus genome contains two open reading frames (ORFs), designated rep and cap, flanked by two inverted terminal repeats. The rep ORF encodes proteins involved in viral replication, and the cap ORF encodes proteins necessary for viral packaging. In rAAV-mediated KO vectors these ORFs are deleted and replaced with a neomycin resistant gene flanked by two homology arms. The stages involved in carrying out rAAV-mediated gene KO include: (i) design and construction of the AAV KO vector; (ii) collecting an infectious rAAV stock; (iii) infecting the appropriate cell line; (iv) screening for homologous recombinants; and (v) iteratively targeting the multiple alleles. It takes at least three months just to target the first allele [6].

Apart from the low efficiency of homologous recombination, a rate-limiting step in gene targeting of human somatic cell lines is assembly of the gene targeting construct. Traditionally, this requires an amplification step to obtain two large homologous fragments of genomic DNA, followed by restriction endonuclease digestion, and then numerous cloning steps. It is an extremely time-consuming process and limited by the available unique restriction enzyme sites in the vector and in the two amplified homologous fragments. Phage-based *Escherichia coli* homologous recombination systems [7-9] have been developed that now make it possible to subclone or modify DNA cloned into plasmids, bacterial artificial chromosomes (BACs), or P1-derived artificial chromosomes (PACs) without the need for restriction enzymes or DNA ligases. However, these recombination systems require long homology arms and usually one can only insert one fragment at the time.

Traditional DNA cloning suffers from several limitations, including poor ligation efficiency, along with a dependence on the availability of unique restriction sites in both the insert and vector. For this reason, many methods regarding directional subcloning have been developed [10,11]. These methods include the use of uracil DNA N-glycosylase [12], T4 DNA polymerase [13], enzymatic assembly [14-16] or exonuclease III (ExoIII) [17] to generate long compatible cohesive ends between the DNA insert and cloning vector. It has also been found that by generating longer cohesive ends, the annealed DNA complex becomes more stable and the ligation reaction can be omitted. This technique has come to be known as ligation-independent cloning (LIC), and has been demonstrated to have a high efficiency. Developing more LIC methods will give the researchers more chioce according to the different situations.

To increase cloning efficiency and ligation of four fragments simultaneously, we developed a one-step LIC method for construction of KO vectors. Using this method, it becomes easy to construct KO vectors in two days. In this paper, we have outlined our strategy for modifying pTK-LoxP-NEO-AAV, and demonstrated successful construction of a SirT1 and HDAC2 KO vector. Once we had made the vectors, we were able to obtain SirT1 KO cells from the colorectal cancer cell line HCT116 and HDAC2 KO cells from the colorectal cancer cell line DLD1.

## Results and discussion

### Construction and characterization of a pAAV-LIC vector

**Figure 1:**
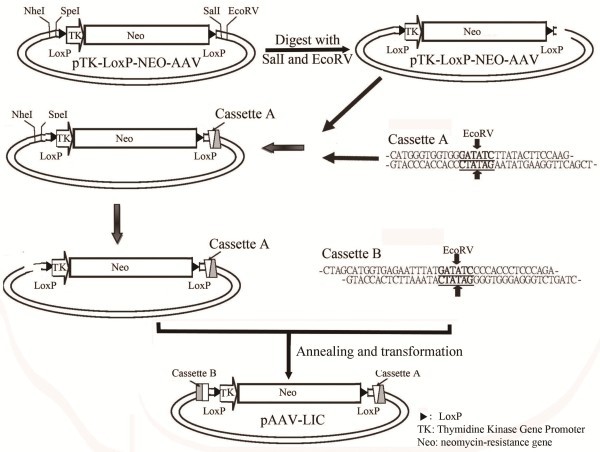
**Schematic depicting the modification of pTK-LoxP-NEO-AAV into pAAV-LIC by insertion of two designed LIC adaptors.** The two LIC adaptors contain *Eco*RV sites and an additional 11–15 bp sequence necessary for LIC cloning. The two *Eco*RV restriction sequences are underlined and an arrow indicates the digestion sites. Left blunt end and right overhang sequences for *Eco*RV and *Sal*I in cassette **A** allow for annealing of the LIC adaptor into the right arm MCS of the pTK-LoxP-NEO-AAV plasmid. Overhang sequences for *Nhe*I and *Spe*I in cassette **B** allow for annealing of the LIC adaptor into the left arm MCS.

Assembly of gene-targeting constructs is a rate-limiting step when targeting genes in human somatic cell lines. Other limitations include multi-step ligations and the availability of unique restriction sites in the insert and vector. To overcome many of these limitations we modified an AAV shuttle vector into a one-step LIC vector. This was achieved by introducing two fixed cassettes into the multiple-cloning site (MCS) of the shuttle vector (Figure 1). The two cassettes involved three elements: sticky ends complementary to the cloning site in pTK-LoxP-NEO-AAV, allowing for insertion of the cassettes; *Eco*RV sites in the middle of the cassette, which could be used to cut the LIC vector into two fragments containing blunt ends; and 14- to 18-mer sequences that could produce long sticky ends when ExoIII was used.

First, cassette A was inserted into pTK-LoxP-NEO-AAV using *Eco*RV and *Sal*I digestion. Because the cassette end did not include the *Eco*RV palindromic sequence, this process restored the *Sal*I site and destroyed the original *Eco*RV site. Following confirmation by sequencing, cassette B was inserted into the vector now containing cassette A. This was facilitated by digestion with *Nhe*I and *Spe*I digestion. The modifications to the vector were again confirmed by sequencing, and we designated the designed vector as pAAV-LIC.

### Construction of a SirT1 and HDAC2 KO vectors

**Figure 2:**
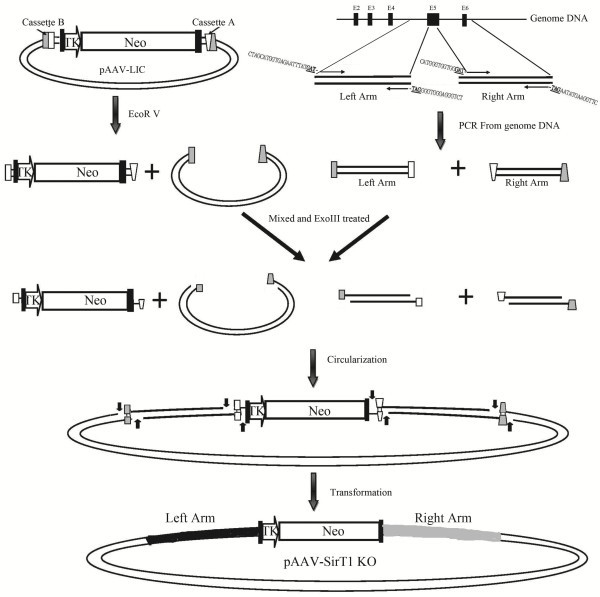
**Construction of a somatic cell KO vector *via *a one-step cloning method.** Cassette A is indicated by two rectangles and cassette B by two echelons. The homologous arms were amplified using PCR with primers containing the 15–18 nt sequence at the 5′ end and sequences specific for the ExoIII-generated overhangs in pAAV-LIC. E2–6: exons 2–6; NEO: neomycin resistance gene; TK: thymidine kinase promoter.

We used pAAV-LIC to construct SirT1 and HDAC2 KO vector by one-step LIC (Figure 2). The pAAV-LIC vector was digested with *Eco*RV, resulting in two fragments (approximately 1 kb and 3.1 kb). These fragments were recovered from agarose gels following electrophoresis and purified using gel extraction kits. The smaller fragment was found to contain the thymidine kinase (TK) promoter, the LoxP sequence and the neomycin ORF. The larger fragment was found to contain the AAV backbone, necessary for plasmid replication in *E. coli*, and for viral packaging.

The two homologous arms for SirT1 and HDAC2 KO vector construction were obtained by polymerase chain reaction (PCR) and purified using gel extraction kits. The PCR primers used were designed to add additional 14- to 18-mer sequences overlapping with the ends of digested vector fragments. Polymerases, including *Taq* polymerase, possess the ability to add dATP at the 3′ ends of PCR products, thereby inhibiting the 3′ exonuclease activity of ExoIII. Therefore we recommend using *pfu* or PrimeStar polymerases when amplifying homologous arms, as they result in amplicons with blunt ends.

The 3′ exonuclease activity of ExoIII is very high at 37°C. To generate 16-mer overhangs at a stable and low rate, it is necessary to optimize reaction temperature and time. According to our experience, to ensure stable cloning, 1 h at 0°C or 30 min at 4°C are both sufficient. Using other conditions proved difficult, with both the number of colonies and the rate of positive transformants acquired highly variable. To produce complementary sticky ends, the purified homologous arms and two vector fragments were mixed and chilled at 0°C for 10 min, then treated with 20 U of ExoIII at 0°C for 1 h. The reaction was terminated using EDTA or by heating to 65°C. After transformation into DH5Î± *E. coli*, enzymatic bacterial DNA repair machinery helped to facilitate ligation endogenously.

**Figure 3:**
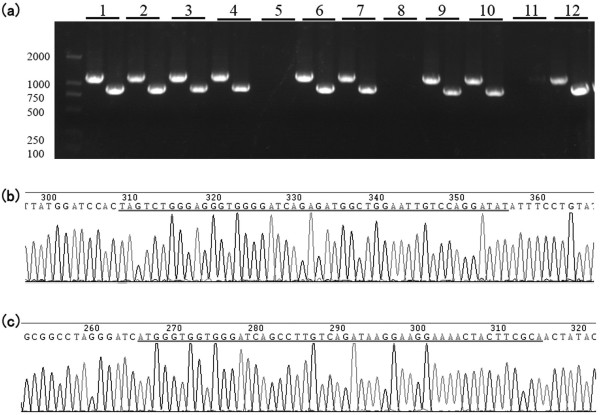
**Colony PCR and sequencing results for the pAAV-SirT1 gene KO plasmid.** (**a**) Colony screening by PCR. Left arm (> 1 kb) and right arm (< 1 kb) positives are shown. (**b**) The sequence in the joint formed by the vector and the left arm of SirT1. The joint sequence is underlined in gray. (**c**) The sequence in the joint formed by the vector and the right arm of SirT1. The joint sequence is underlined in gray.

Colony PCR and sequencing was used to verify the positive transformants. Screening by PCR showed that 9 of the 12 colonies analyzed were positive transformants (Figure 3a). Sequencing results further confirmed the PCR results (Figure 3b and c). The resulting KO vector was designated as the AAV-SirT1 KO vector. Also we got the AAV-HDAC2 KO vector.

### Use of gene targeting to generate SirT1-null HCT116 cells and HDAC2-null DLD1 cells

**Figure 4:**
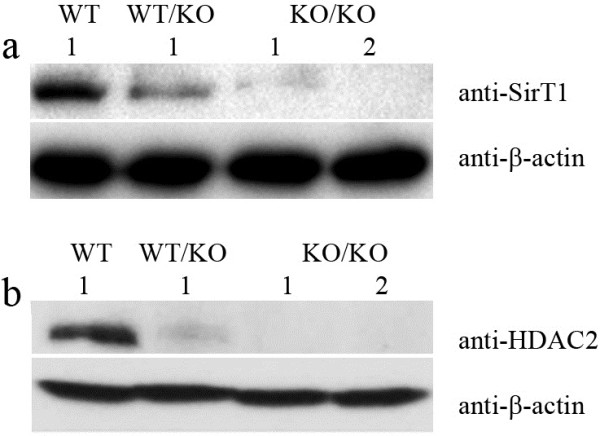
**KO cell lines were confirmed by western blot.** (**a**) Western blots of wild-type, heterozygous and homozygous SirT1 KO cell lines from HCT116 cells using anti-SirT1 and anti-β-actin antibodies. WT indicates parental cells, WT/KO indicates SirT1 heterozygous KO cells, KO/KO indicates SirT1 homozygous KO cells. (**b**) Western blots of wild-type and homozygous HDAC2 KO cell lines from DLD1 cells using anti-HDAC2 and anti-β-actin antibodies. WT indicates parental cells, WT/KO indicates HDAC2 heterozygous KO cells, KO/KO indicates HDAC2 homozygous KO cells.

To confirm its ability to be used in the development of KO cell lines, the targeting AAVs were packaged in 293T cells by transfecting equal amounts of the SirT1 KO targeting vector targeting vector, pHelper and pRC. Then HCT116 cells were infected with the SirT1 targeting viruses and selected with geneticin. After two weeks, the neomycin-resistant clones were screened for homologous recombination by genomic PCR. We screened 100 clones and obtained 4 positives. To remove the neomycin-resistant gene, two of the correctly targeted clones were infected with adenoviruses expressing Cre-recombinase. Using genomic PCR, we screened 24 clones, with 10 of them found to be positives containing an additional 250 bp fragment in which the LoxP site was inserted. Up until this point we had succeeded in obtaining heterozygous KO clones. To delete both alleles, heterozygous KO clones were infected with the same targeting virus and the neomycin resistance gene was excised. Finally we obtained homozygous KO clones and the KO cell lines were confirmed by western blot (Figure 4a). Use the same protocol, HDAC2 targeting viruses were made and DLD1 cells were infected with the HDAC2 targeting viruses. Also we obtained HDAC2-null DLD1 cells and confirmed by western blot (Figure 4b).

## Conclusions

As outlined above, we successfully constructed the AAV-SirT1 and AAV-HDAC2 KO vectors and obtained SirT1 and HDAC2 KO cell lines. Compared with the traditional ‘digestion-ligation’ vector construction method, our approach is not limited by the availability of restriction sites; therefore, somatic cell gene KO vectors can be easily constructed within 2–3 days. Additionally, our method does not require the use of any single specific restriction enzyme, and has great potential for the high-throughput construction of gene KO vectors. However, it also has some limitations, such as the potential errors can be produced during amplification by PCR and it need high efficiency competent cells for the transformation.

## Methods

### Materials and bacterial strains

Restriction enzymes, *Pfu* polymerase, ExoIII and DNA ligation kits were purchased from Takara (Dalian, China). Kits for plasmid minipreps, DNA purification and genome extraction were purchased from Tiangen (Beijing, China). Primers were purchased from Genscript (Nanjing, China). The *E. coli* DH5Î± strain was used for plasmid preparation and all restriction-based cloning.

### Cell culture

HEK-293T cells were cultured in Dulbecco’s modified Eagle’s medium (DMEM) containing 10% fetal bovine serum (FBS) and 100 units/ml penicillin-streptomycin. The colorectal cancer cell line, HCT116, was cultured in McCoy’s 5A containing 10% FBS and 100 units/ml penicillin-streptomycin. McCoy’s 5A medium was purchased from AppliChem (Seajet Scientific Co., Ltd., Beijing, CHINA). All other cell culture reagents were obtained from Life Technologies Corporation (Shanghai, CHINA).

### Construction of the modified AAV shuttle vector

The AAV shuttle vector, pTK-LoxP-NEO-AAV, was modified to produce the pAAV-LIC vector. Two pairs of unique primers were designed for creating ‘LIC zones’. These primer pairs were cassette A (sense, 5′-CAT GGG TGG TGG GAT ATC TTA TAC TTC CAA G-3′; and antisense, 5′-TCG ACT TGG AAG TAT AAG ATA TCC CAC CAC CCA TG-3′) and cassette B (sense, 5′-CTA GCA TGG TGA GAA TTT ATG ATA TCC CCA CCC TCC CAG A-3′; and antisense, 5′-CTA GTC TGG GAG GGT GGG GAT ATC ATA AAT TCT CAC CAT G-3′). The underlined sequence indicates the recognition site for *Eco*RV.

Primers were diluted to 5 mM with double-distilled water. Sense and antisense primers (3 μl of each) for cassette A or cassette B were mixed with 3 μl of buffer 3 (NEB) and 21 μl of sterile water. The reaction was incubated at 94°C for 10 min, then cooled slowly to room temperature. Cassette A or B fusion products were inserted into the digested pTK-LoxP-NEO-AAV vector using DNA ligation kits. Recombinants were verified by restriction analysis and sequencing.

### General primers for AAV gene-targeting vectors

The homologous arm is around 1 kb, and we designed primers using primer3 ( http://frodo.wi.mit.edu/primer3/). To insert arms into the pAAV-LIC backbone, the primers needed to contain complementary sequences to the ExoIII-generated overhangs in pAAV-LIC. The designed and synthesized primers were Left Arm Sense (5′-ATG GTG AGA ATT TAT GAT-3′), Left Arm Antisense (5′-TGG GAG GGT GGG GAT-3′), Right Arm Sense (5′-ATG GGT GGT GGG AT-3′), and Right Arm Antisense (5′-TTG GAA GTA TAA GAT-3′).

### Construction of the SirT1 AAV KO vector

Plasmid pAAV-LIC (2 μg) was subjected to overnight digestion with *Eco*RV in a 50 μl reaction at 37°C. The two fragments produced were purified using a gel extraction kit and diluted to 50 ng/μl in TE buffer (pH8.0). Two homologous arms were amplified from genomic DNA extracted from HCT116 cells. Two pairs of unique primers were designed: (a) SirT1, left arm, forward (5′-ATGGTGAGAATTTATGATCGCCTGTTTGTATCCTTCCTGAC-3′) and reverse (5′-TGGGAGGGTGGGGATCAGAGATGGCTGGAATTGTCC–3′); and (b) SirT1, right arm, forward (5′–ATGGGTGGTGGGATCAGCCTTGTCAGATAAGGAAG–3′) and reverse (5′–TTGGAAGTATAAGATCACCTTAGCTCGGCAGTTCTT–3′); (c) HDAC2, left arm, forward (5′- ATGGTGAGAATTTATGATAGCCAGCAGCATTGCTGCAG −3′) and reverse (5′–TGGGAGGGTGGGGATAGCCGCGGAACCCAGCGCC −3′); and (d) HDAC2, right arm, forward (5′–ATGGGTGGTGGGATAAGTCTGCTACTACTACGAC −3′) and reverse (5′–TTGGAAGTATAAGATCCCCAAAGCAGGGTCTAT −3′). The underlined sequences indicate the designed primers from the SirT1 genome DNA or HDAC2 genome DNA. The PCRs were performed using *pfu* polymerase, with amplicons electrophoresed and then recovered by gel extraction kits. Recovered fragments were diluted to 100 ng/μl in TE buffer.

The vector fragments and amplicons were mixed in one reaction tube. The large fragment (1 μl), small fragment (2 μl), left arm (2 μl) and right arm (2 μl) were mixed with 1 μl of 10× ExoIII buffer and 1 μl of sterile water. The tube was pre-chilled on ice for 2 min, and then 1 μl of ExoIII (20 units) was added to the tube and gently mixed. After a 1 h incubation at 0°C, 1 μl of 0.5 M EDTA was added to stop the reaction. The enzyme was inactivated at 65°C for 5 min. After cooling, the mixture was transformed into *E. coli* DH5Î± and cultured at 37°C overnight. Positive transformants were identified by colony PCR and sequencing.

### Somatic cell gene targeting

Somatic cell gene targeting was conducted as described previously [5]. Briefly, the targeting AAVs were packaged in 293T cells by transfecting equal amounts of SirT1 or HDAC2 KO targeting vector, pHelper and pRC plasmids (1 mg each). After 72 h, scraped the transfected cells and suspended the cells into sterile phosphate-buffered saline, then freezed and thawed the pellet three cycles, finally spined the lysate to remove cell debris and divided the supernatant containing rAAV into several aliquots and frozen at-80°C. HCT116 or DLD1 cells were infected with the SirT1 or HDAC2 targeting viruses and selected with geneticin for two weeks. The geneticin-resistant clones were then screened for homologous recombination by genomic PCR with primers derived from the neomycin resistance gene (5′-GTTGTGCCCAGTCATAGCCG-3′) and the upstream region of the left homologous arm (SirT1: 5′-GAAAGTTCCCCACATCTGCT-3′; HDAC2: 5′-TGCTCCAATCTTC CAGTGTCT-3′). Positive clones were confirmed by genomic PCR, with primers derived from the neomycin resistance gene (5′-TCTGGATTCATCGACTGTGG-3′) and the downstream region of the right homologous arm (SirT1: 5′-TCACTCCTCCAGGGCTAAAA-3′; HDAC2 5′-CACCTAGGAACAGCCTTTGC-3′). Correctly targeted clones were infected with adenoviruses expressing Cre-recombinase to delete the selectable drug marker. To select clones with successful deletion of the selectable drug marker, genomic PCR was employed to amplify an approximate 250 bp fragment in which the LoxP site was inserted, using specific primers (SirT1:5′-TAGGTGTGTGTCGCATCCAT-3′ and 5′-CCTGTTCCAGCGTGTCTATGT-3′; HDAC2:5′- CATGGCGTACAGTCAAGGAG-3′ and 5′-CAAATGTCGGTCCCTCCTC-3′). The heterozygous KO clones were infected with the same targeting virus to target the second allele and the neomycin resistance gene was excised as described earlier. Final confirmation in the generation of the KO cell lines was done using western blotting.

## Competing interests

The authors declare they have no competing interests.

## Authors’ contributions

RD conceived the project and designed the experiments along with YL. YL, HZ and SL performed the laboratory work and analyzed the results. XZ and R D wrote the manuscript. All authors read and approved the final manuscript.
